# Thalidomide and neurotrophism

**DOI:** 10.1007/s00256-018-3086-2

**Published:** 2018-10-19

**Authors:** Judith R. Soper, S. Fiona Bonar, Dudley J. O’Sullivan, Janet McCredie, Hans-Georg Willert

**Affiliations:** 1 Diagnostic Radiology, Royal Prince Alfred Hospital, Missenden Rd, Camperdown, Sydney, NSW Australia; 2 Specialist Magnetic Resonance Imaging Carillon Ave Newtown, Sydney, NSW Australia; 3 Douglass Hanly Moir, Giffnock Ave Macquarie Park, Sydney, NSW Australia; 4 Anatomical Pathology Royal Prince Alfred Hospital, Missenden Rd Camperdown Sydney, Sydney, NSW Australia; 5 Neurology, St Vincent’s Hospital, Darlinghurst, NSW Australia; 6 Diagnostic Radiology, Faculty of Medicine University of Sydney, Sydney, NSW Australia; 7 Orthopaedic Surgery, Georg-August Universität, Göttingen, Germany

**Keywords:** Thalidomide, Neurotrophism, Embryonic sensory neuropathy, Quantitative neuropathology, Embryonic sensorineural osteoarthropathy

## Abstract

**Background:**

Following the thalidomide disaster (1958–62), Henkel and Willert analysed the pattern of dysmelia in the long bones (J Bone Joint Surg Br. 51:399–414, 1969) and the extremities, Willert and Henkel (Z Orthop Ihre Grenzgeb. 107:663–75, 1970). Willert’s material from deformed extremities is re-examined here asking “How does thalidomide reduce the skeleton?”

**Materials and methods:**

We reviewed the original data collection of Willert and Henkel (Z Orthop Ihre Grenzgeb. 107:663–75, 1970), comprising musculoskeletal histology slides from 30 children affected by thalidomide with radiographs of hands (19 cases) and feet (4 cases).

**Results:**

All original observations by Willert and Henkel (Z Orthop Ihre Grenzgeb. 107:663–75, 1970), were verified. Radial rays of the hand disappeared early, but the foot was spared until late. Radiology confirms that bone reduction in the hand (aplasia or hypoplasia in the thumb and index finger) coincides with sensory segmental nerve C6. In the foot, reduction of the toes is rare, but mesenchymal excess (polydactyly) occurs in the hallux (L5 sclerotome), usually associated with absent tibia (L4 sclerotome). Histology confirms skeletal mesenchymal components to be unremarkable, contrasting with grossly abnormal bony architecture, a striking discordance between microscopic and macroscopic findings. No necrosis or vascular pathology was seen.

**Conclusion:**

The basic lesion was an abnormal quantity rather than quality of mesenchyme. Cell populations result from cellular proliferation, controlled in early limb bud formation by neurotrophism. Thalidomide is a known sensory neurotoxin in adults. In the embryo, sensorineural injury alters neurotrophism, causing increased or diminished cell proliferation in undifferentiated mesenchyme. Differentiation into normal cartilage occurs later, but within an altered mesenchymal mass. Reduction or excess deformity results, with normal histology, a significant finding. The primary pathological condition is not in the skeleton, but in the nerves.

## Introduction

**Fig. 1:**
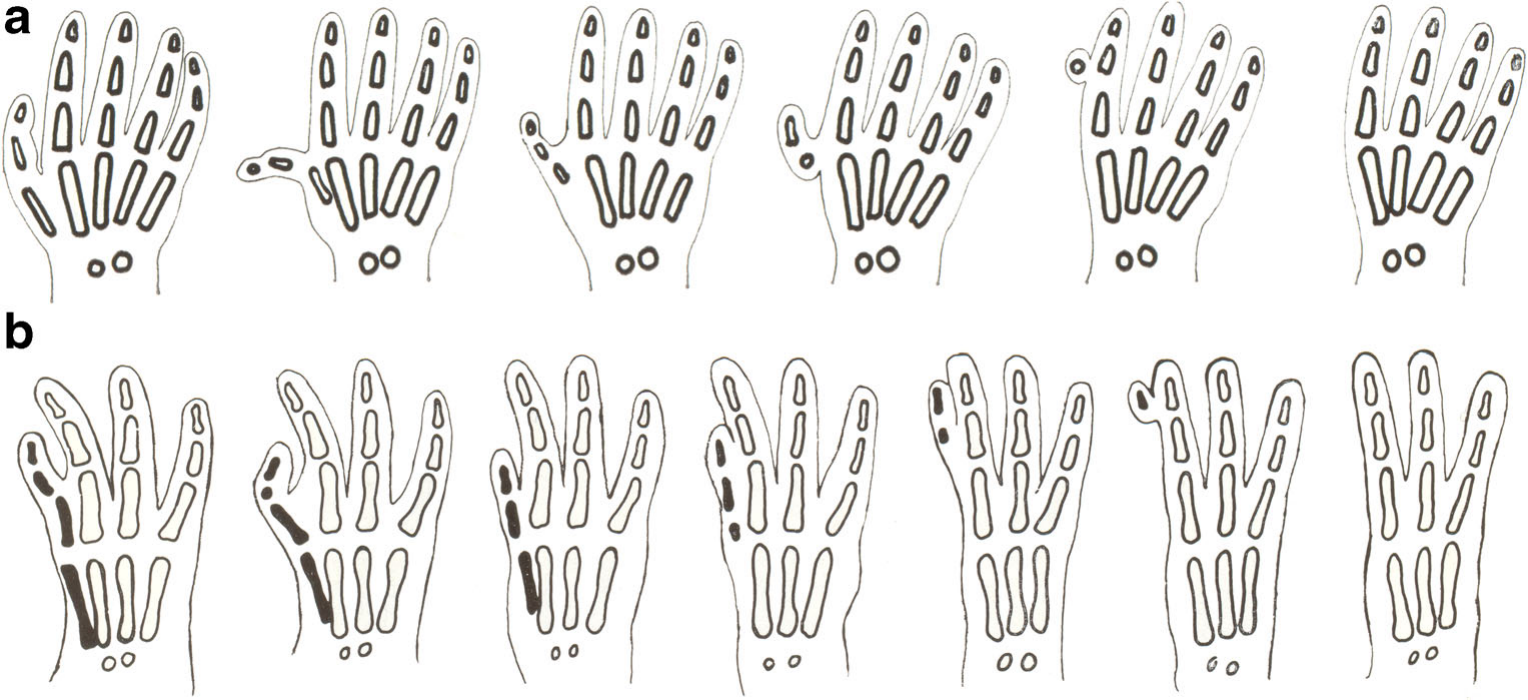
**a** Thumb reduction in thalidomide embryopathy. As the severity of damage increases, the thumb and first metacarpal are reduced from normal to total absence, through hypoplastic and triphalangeal morphology to pedunculated remnants, and then no thumb at all [2]. A non-functional pedunculated digital remnant was usually surgically removed in infancy. **b** Index finger reduction from normal to nil. The second metacarpal disappears before the phalanges. Two or three phalanges may be reduced in size or fused to the adjacent third ray by the syndactyly of soft tissues or by a narrow pedicle (flail digit) [2]

**Fig. 2:**
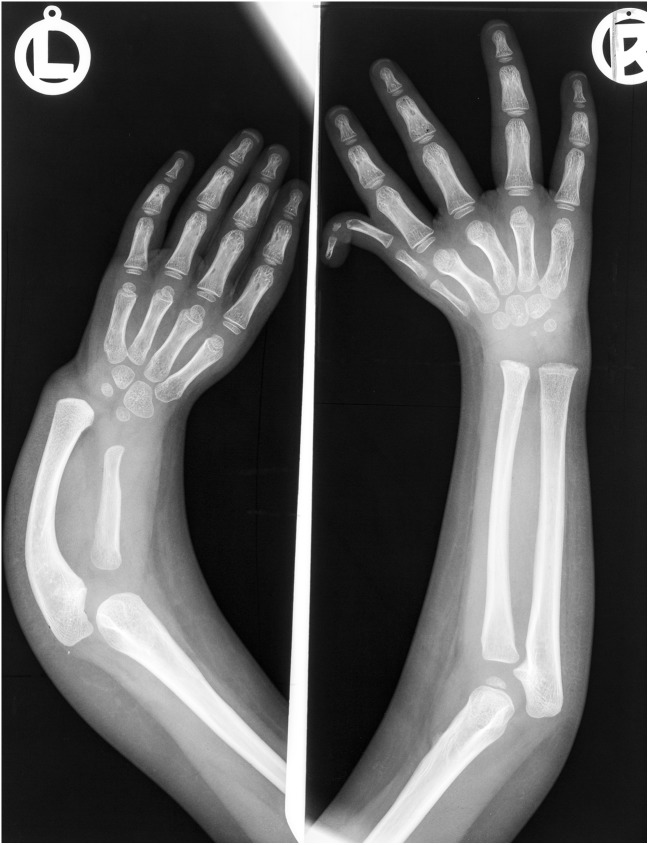
Forearms of a child affected by thalidomide. Absent left thumb, short hypoplastic radius, compensatory curved ulna. Hypoplastic right triphalangeal thumb with short middle phalanx. The first metacarpal is duplicated. Damage to sclerotome C6 bilaterally, but more severe on the left

The thalidomide catastrophe (1958–1962) generated an epidemic of longitudinal reduction deformities of the limbs (dysmelia) in all countries where the drug was marketed, thousands in West Germany, home of the drug. Henkel and Willert’s first paper (1969), published in English, defined the pattern and principles of skeletal reduction in 287 cases (Figs. 1, 2 and see Fig. 5d) [1]. Their second paper (1970) in German sought underlying histopathology in another 30 cases involving hands and feet [2], failing to find it.

Willert’s 30 digital cases are unique: all include skeletal histology. Translated into English, the findings are discussed, applying accepted concepts from neurology and regeneration biology. The distribution of limb deficiency coincided exactly with segmental sensory nerve supply [3]. Thalidomide is a clinically proven sensory neurotoxin [4–9]; yet, its mode of teratogenic action is still debated. Sensory neurotrophism stimulates proliferation of undifferentiated mesenchyme [10–19]. Does the reduction or addition of mesenchymal mass in the extremities indicate damaged neurotrophism, secondary to the primary damage to embryonic sensory nerves caused by thalidomide?

## Materials and methods

Willert’s well-catalogued material from amputations or autopsies on 30 thalidomide cases was assessed:

1. Haematoxylin and eosin-stained sections of paraffin-embedded skeletal tissue.
2. Radiographs from 23 of the 30 cases: 19 hand and 4 feet abnormalities.
3. Pathology slides only were available in 7 of the 30 cases. Whether these represented hand or foot bones and joints was unclear.

The German paper was translated into English, conserving scientific observations.

Histopathology slides and radiographs were re-examined. Our new discussion of the teratogenic mechanism of thalidomide applied principles from neurology and regeneration biology to these data.

## Results

We verified the original findings [2]. Changes in the extremities simulated those in long bones [1]. Abnormalities in radial fingers or tibial toes appeared alone or combined with reduction deformities in the radius and tibia [1]. Fingers commonly showed reductions: a short middle phalanx, or a reduced or absent thumb, with frequent triphalangism, but only occasional polydactyly. Toe reductions were rare, but additions were common: triphalangeal hallux or polydactyly.

Willert and Henkel analysed the action of thalidomide as two opposing processes that alter the amount of undifferentiated mesenchyme forming the extremity, causing either reduction or addition of mesenchymal mass.

### Mesenchymal reduction

#### Radiology

**Fig. 3:**
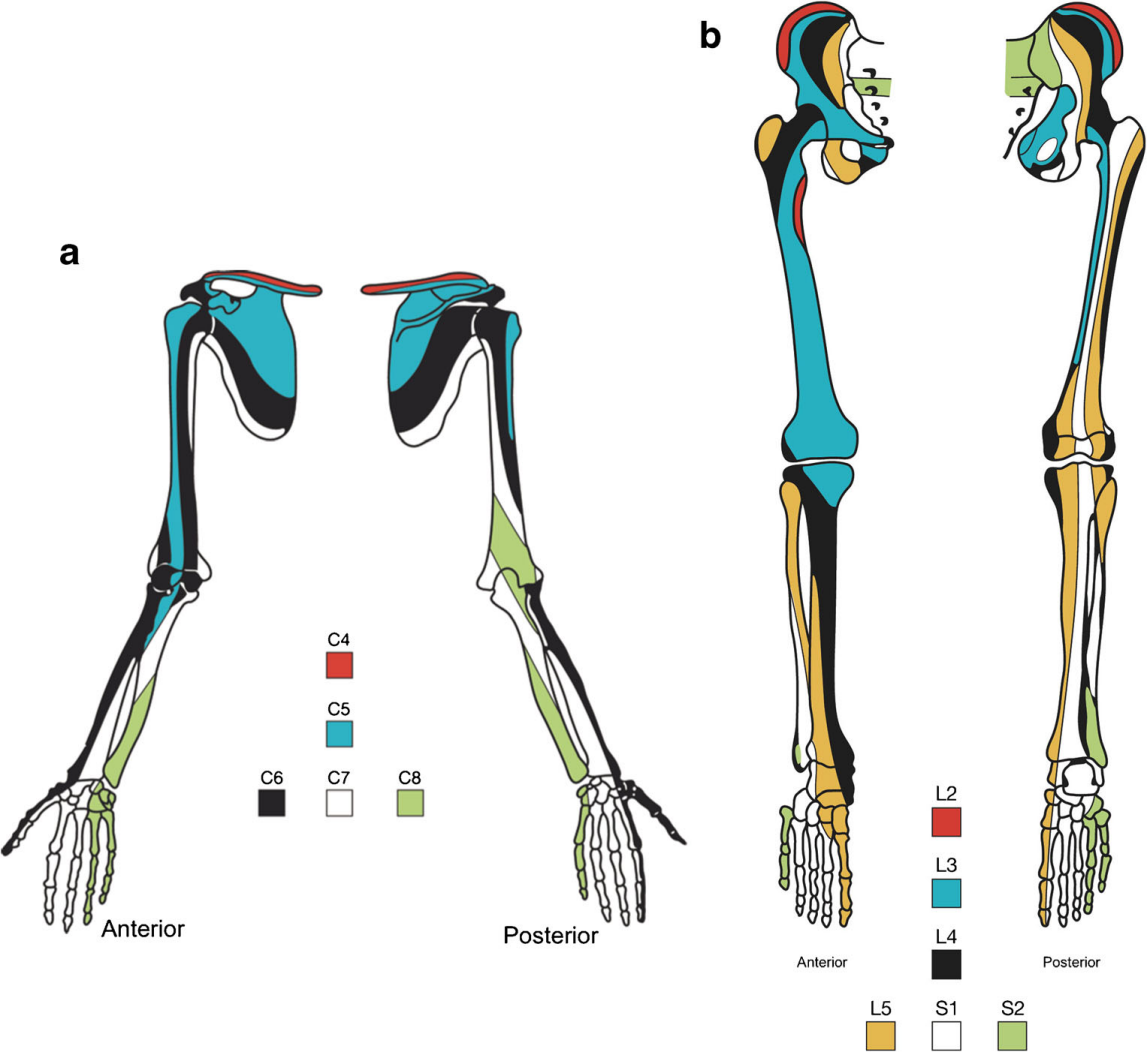
**a** Sclerotomes of the upper limb: cervical segmental sensory nerve supply to the skeleton, derived from studies of referred pain [20]. A sclerotome is defined as a band of skeletal structures supplied by one spinal segmental sensory nerve. It is the somatic equivalent of a dermatome. It crosses through soft tissues, bones and joints, irrespective of the usual anatomical structures such as joint spaces. **b** Sclerotomes of the lower limb: lumbar and sacral sensory nerve supply to the lower limbs [20], incorporating the repositioning of the hallux from a superior to medial position during development of leg length in utero, demonstrated by medial twisting of sclerotomes. L4 was most vulnerable to thalidomide with sclerotome deletion; L5 could show addition/polydactyly

Mesenchymal reduction occurs predominantly within the sixth cervical nerve supply (Fig. 3a) [3, 4].

##### Thumb

The reduction process commenced with the thumb, proceeding with increasing severity (Figs. 1a, 2). Decreasing mass narrowed the shape and size of the phalanges and first metacarpal. Reduction began proximally, progressing distally. The first metacarpal was most affected: the proximal part progressively disappeared and this was the first bone to vanish. Finally, a minute vestige of the phalanx was left attached to the radial side of the index finger (Fig. 1a).

##### Index finger

Again, underdevelopment of the metacarpal and phalanges particularly affected the middle phalanx, progressing to complete disappearance, forming a finger with only two of the three phalanges. Subsequent reduction followed the thumb pattern: skeletal loss progressed proximodistally with the second metacarpal disappearing first. Finally, vestigial phalanges were attached to the third finger (Fig. 1b).

##### Third finger

The same pattern occurred. Soft-tissue fusions appeared in interdigital spaces between affected rays.

#### Pathology

**Fig. 4:**
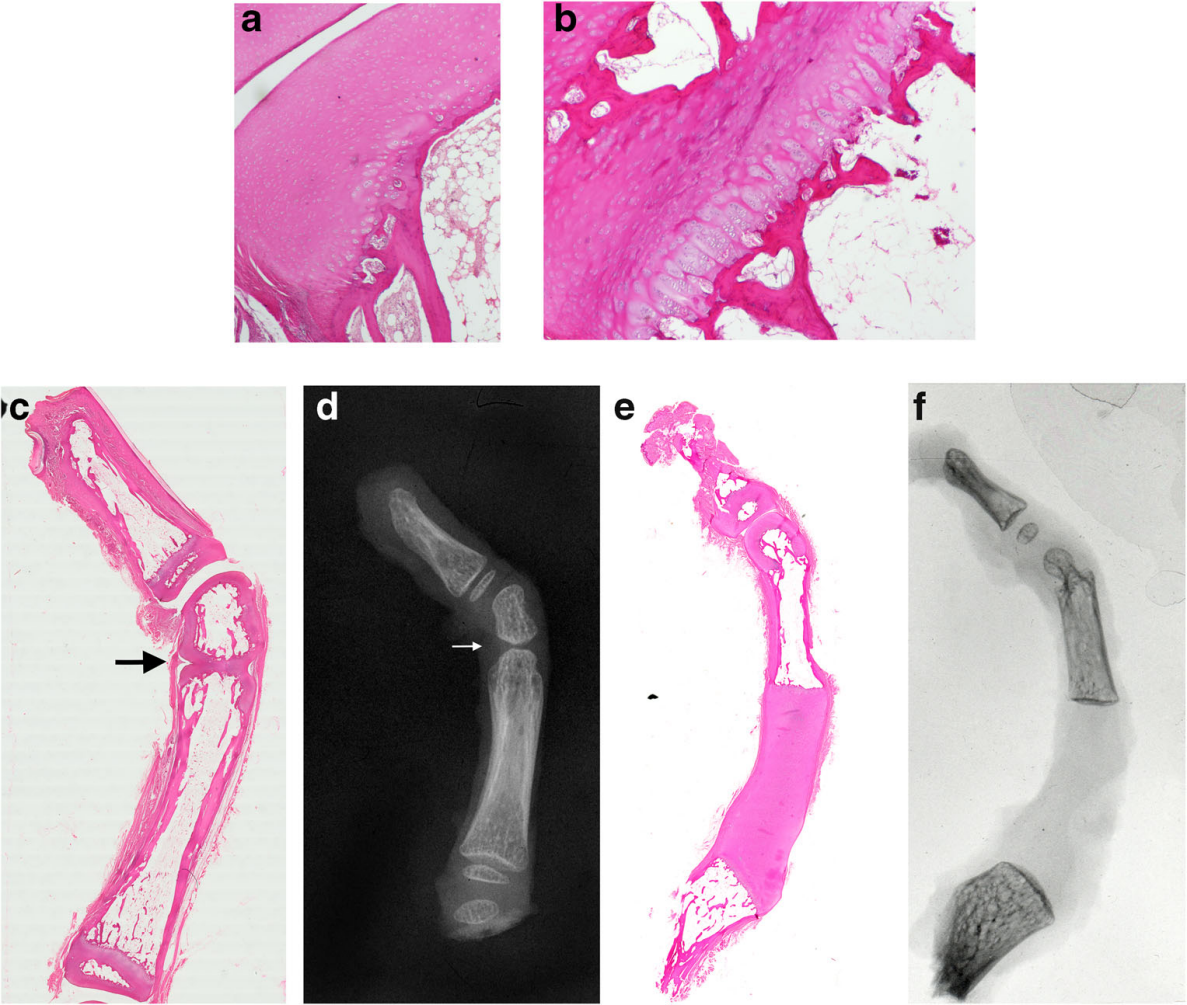
**a** Normal hyaline cartilage and bony components at low power. **b** Epiphyseal plates were unremarkable with reserve, proliferative and hypertrophic zones with endochondral ossification. **c** Wholemount section and **d** specimen radiograph showing a small middle phalanx and fused incomplete proximal interphalangeal joint replaced by a notch (*black arrow*). A cleft is visible on the radiograph (*white arrow*). **e** In some cases, complete lack of ossification of the proximal phalanx was present, **f** illustrating apparent discontinuity of the structures on imaging

In the affected digits, the microscopic appearance of the cells and mesenchymal components was normal, but the sizes and shapes of the bones were abnormal. The characteristics of the reduction process, observed by Willert and Henkel, were confirmed:

1. Deficient mesenchymal tissue: Macroscopically, although the digits had short middle phalanges, the component cells and matrix, including cartilage and bone, were microscopically normal. All of the cellular components were as expected in otherwise normal digits. There was no evidence of vascular pathological conditions, cellular necrosis or previous haemorrhage to explain the observed reduction in tissue mass. Deposit accumulation, a feature of storage disorders, was not present (Fig. 4a, b).

Underdevelopment of the middle phalanx in the process of reduction deformity is characterised by three morphological criteria: reduction in mass and loss of normal shape of the middle phalanx; absence of joint cleavage and impairment of joint development in less severe cases and absence of joint development in more severe cases, including fusion of a rudimentary phalanx to its neighbour (synchondrosis or synostosis); delayed ossification of hyaline cartilage. Combinations of these three criteria occur.

2. Loss of normal shape: Together with a reduction in mass, a loss of the normal shape of the bone was observed. The normal structure of diaphysis, metaphysis and epiphysis was absent within these plump middle phalanges. They ossified from a single ossification centre, without formation of a bony perichondrial splint that normally identifies the diaphysis. The epiphyseal plate was usually absent (Fig. 4c, d).
3. Joint angulation, fusion, notches. Interphalangeal joints of reduced bones showed angulation of bone ends with later bending or deviation of the growing digit, frequently uncorrectable. Malformations of articular surfaces were noted at adjacent, less affected joints such as the heads of the first phalanges and metacarpals. Skeletal remnants were frequently united to a neighbouring, less damaged bone (Figs. 1a, b) by cartilage during infancy (synchondrosis); later, endochondral ossification proceeded, to be replaced by a bony bridge (synostosis) when the fusion became visible radiographically. Sometimes the vestige of a joint cleft was seen as a lateral notch or notches at the site where the joint would have been. Remnants of structures belonging to the joint capsule and ligaments could be found here (Fig. 4c, d). The cells and matrix of hyaline cartilage fanned out radially from the bottom of the cleft.
4. Delayed ossification. Commencement of endochondral ossification was delayed. In infancy, the various damaged skeletal elements were present as hyaline cartilage. The middle phalanx of a malformed index finger may contain only a small ossification centre when endochondral ossification should be complete.
5. Normal histology but disturbed maturation. A lack of ossification of hyaline cartilage was also evident in malformed thumbs. At light microscopy no structural change could be found in the cartilage to explain these phenomena, but delayed ossification suggests a disturbance in cartilage maturation, which meant that radiographs in early childhood revealed only part of the picture, as malformations in unossified cartilage remained hidden. Ossification had often not even commenced when an infant was first X-rayed. Such radiographic “empty spaces” represent unossified cartilage (Fig. 4e, f).

### Mesenchymal addition

#### Pathology

Characterised by excess rather than insufficient mesenchyme, the main form is triphalangism of the thumb or hallux, sometimes associated with duplication and syndactyly. Like reduction, the supernumerary skeletal elements show various stages of severity. All mesenchymal elements have an orderly arrangement mimicking a normal digit and no specific microscopic anomaly was identified.

**Fig. 5:**
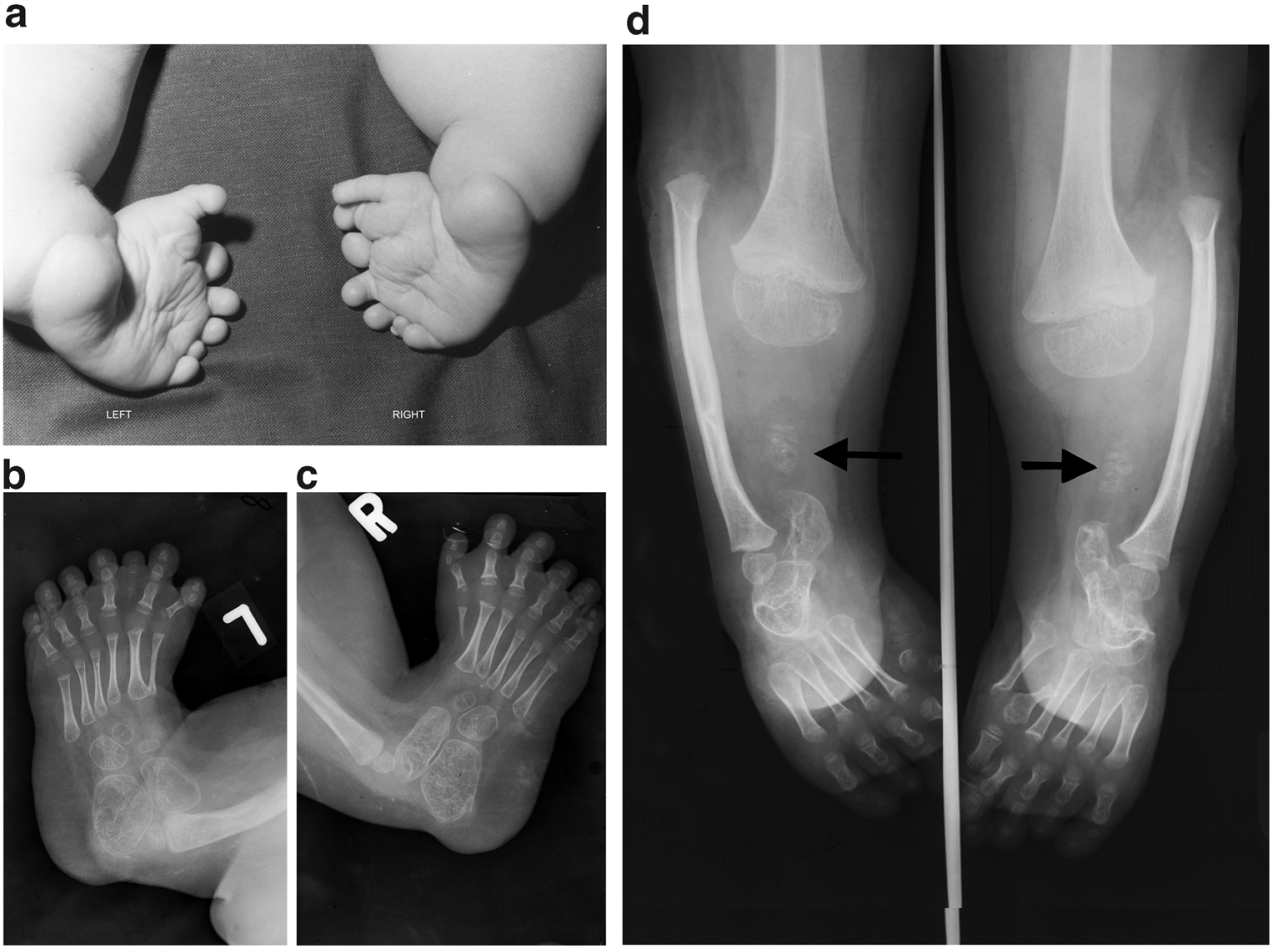
**a** Photograph shows infant with bilateral polydactyly of the toes. **b** Left foot with duplicated first metatarsal and triphalangeal hallux. An additional partial digit is seen in the next webspace. Polydactyly in the L5 sclerotome. Single long bone in the lower leg. **c** Right foot with duplication of the distal first metatarsal and triphalangeal hallux. Single long bone in the lower leg. Partial polydactyly in the L5 distribution. **d** Both lower legs of another patient. The right side shows a small bone representing residual tibia of the distal L3 sclerotome (*black arrows*). L4 sclerotome is absent. Wide fibula is due to fusion of the distal tibia and fibula (sometimes called “tibula”). Four metatarsals with only the head of the first metatarsal present. The left side shows partial resection of a duplicated hallux with residual partial duplication of the first metatarsal

#### Radiology

##### Triphalangeal thumb or hallux

Serial stages culminated in a fully developed, but frequently weak triphalangeal thumb. The additional phalanx was anatomically normal, complete with basal epiphysis. Ossification may reveal a supernumerary distal epiphysis (Fig. 4e, f). In a less well-developed middle phalanx, the basal epiphysis disappeared first. The additional phalanx was smaller than normal, varying down to a mere bony rudiment. The corresponding first metacarpal or metatarsal may be hypoplastic (Fig. 2).

##### Supernumerary toes: polydactyly

Abnormalities of the toes are much rarer than those of the thumb and index finger. The original material was insufficient to determine whether variants of triphalangism in the hallux and thumb corresponded exactly, but the same characteristics of triphalangeal thumbs are observed in supernumerary toes, distributed within the fifth lumbar nerve supply (Figs. 3b, 5d). Underdeveloped middle phalanges in supernumerary toes mimic the short middle phalanx in triphalangeal thumbs (Fig. 5).

## Discussion

Congenital limb reduction is rare and case series are few. Before thalidomide, congenital digital reductions were sporadic and without identifiable environmental, traumatic or familial association [21]. Rare clinical syndromes with specific gene mutations occur [22, 23]. Mutations in major evolutionary pathways, or vascular aetiology may apply in rare instances [23–26], but neither can explain the action of thalidomide. Cell necrosis can reduce mesenchymal mass [25], but no evidence of cell necrosis was seen in our material. Laboratory studies of chick limb development concentrate on molecular phenomena related to control of cell migration, proliferation and differentiation in limb mesenchyme by “patterning” genes and transcription factors [22, 23, 27, 28]. Nerves are never mentioned, yet the innervation of undifferentiated limb buds is proved [29]. Despite much molecular research and debatable claims [25–27, 30, 31], the teratogenic mechanism of thalidomide remains controversial [27, 28, 30–33].

Our review confirms Willert and Henkel’s original observation [2] that the outstanding histological feature of thalidomide is variable tissue mass; either reduced or increased quantity of otherwise unremarkable mesenchymal components.

The subject of the quantity of undifferentiated mesenchyme in the immature human limb bud has rarely been addressed, yet it is pivotal to the teratogenic process underlying these defects. The question is: how does thalidomide cause deficient or excess mesenchymal mass?

Damage to a simple, known mechanism—neurotrophism—answers this and explains the findings. Neurotrophism is growth (cell proliferation) stimulated by nerves [10–18]. That neurotrophism exists throughout the animal kingdom is well established, albeit less obvious in humans than in lower vertebrates that can regenerate lost limbs [19]. Amphibia require sensory innervation of the limb for regeneration [10–13]. In undifferentiated mesenchyme of amphibian limb blastema, prolific mitotic figures accompany the initial increase in mass, long before tissue differentiation [15, 19].

In mammalian embryonic limb buds, electron microscopy reveals innumerable axons ramifying within undifferentiated mesenchyme, predating any other tissue differentiation [29]. In humans, the longitudinal pattern of skeletal depletion caused by thalidomide coincides [3, 20, 34] with maps of segmental sensory nerve supply to the skeleton [20], placing the thalidomide lesion in the embryonic neural crest [4, 34, 35], progenitor of sensory and autonomic nervous systems [22].

**Fig. 6:**
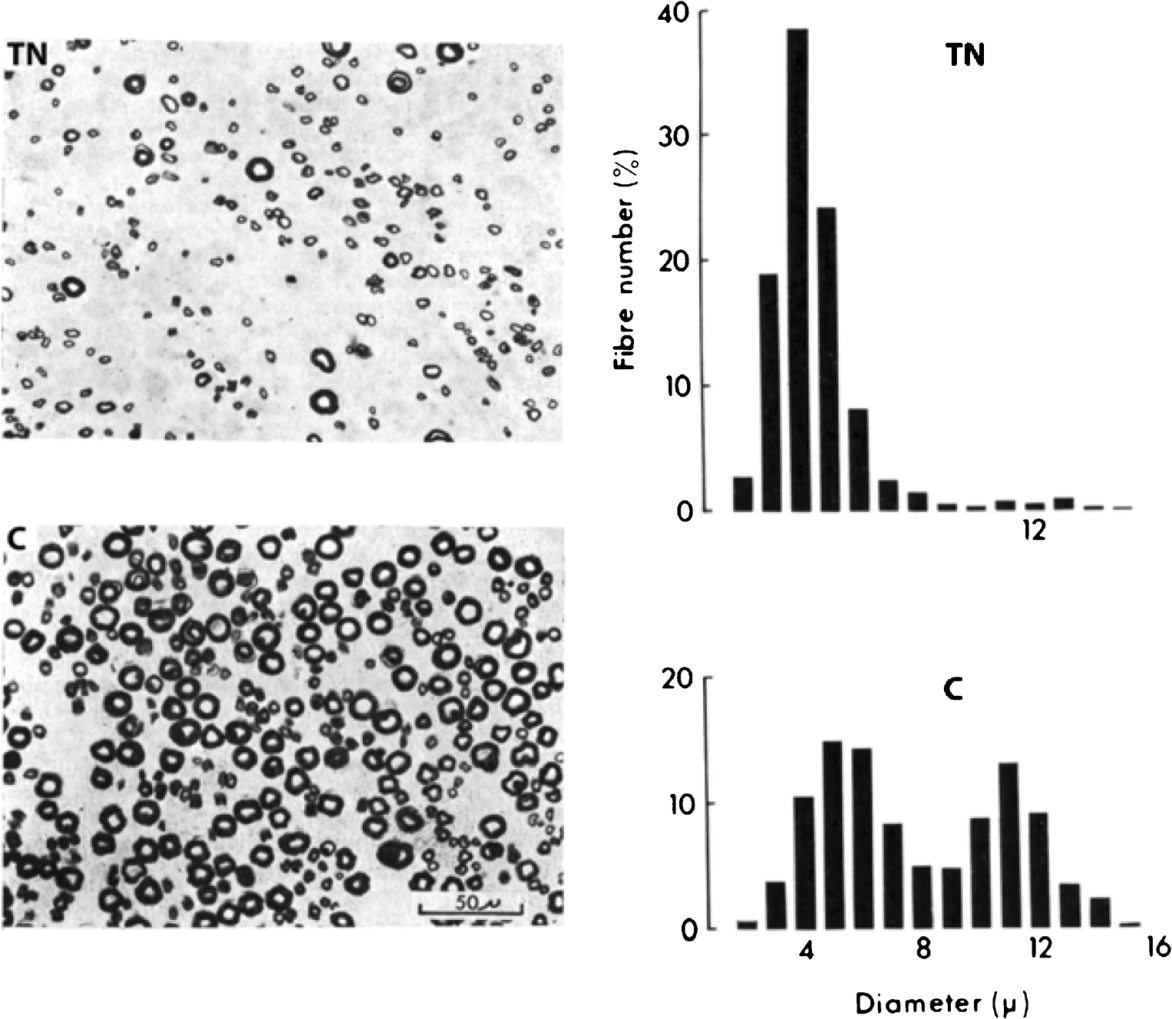
*TN* Portion of transverse section of the sural nerve from a woman with thalidomide neuropathy of 5 years’ duration, microscopy and histogram of fibre diameters. The large diameter fibres have been destroyed [9]. *C* Normal control subject of the same age [9]. Normal bimodal histogram of fibre diameters

Thalidomide targets sensory nerves in adults, causing intractable sensory peripheral neuropathy [5–9]. Figure 6 shows the sural (sensory) nerve of a woman with thalidomide neuropathy of 5 years’ duration [9]. Large diameter axons are dying or dead. It is an axiom in neurology that damaged axons fail to function: sensation fails in the adult; neurotrophic function fails in the embryo. If, in the embryo, neurotrophic stimulation of cell division by a segmental sensory nerve is reduced, the mass of undifferentiated mesenchyme generated by that nerve is reduced. Nerve/neurotrophic damage thus results in segmental reduction of mesenchymal mass, and at birth, longitudinal reduction deformity in the skeleton.

**Fig. 7:**
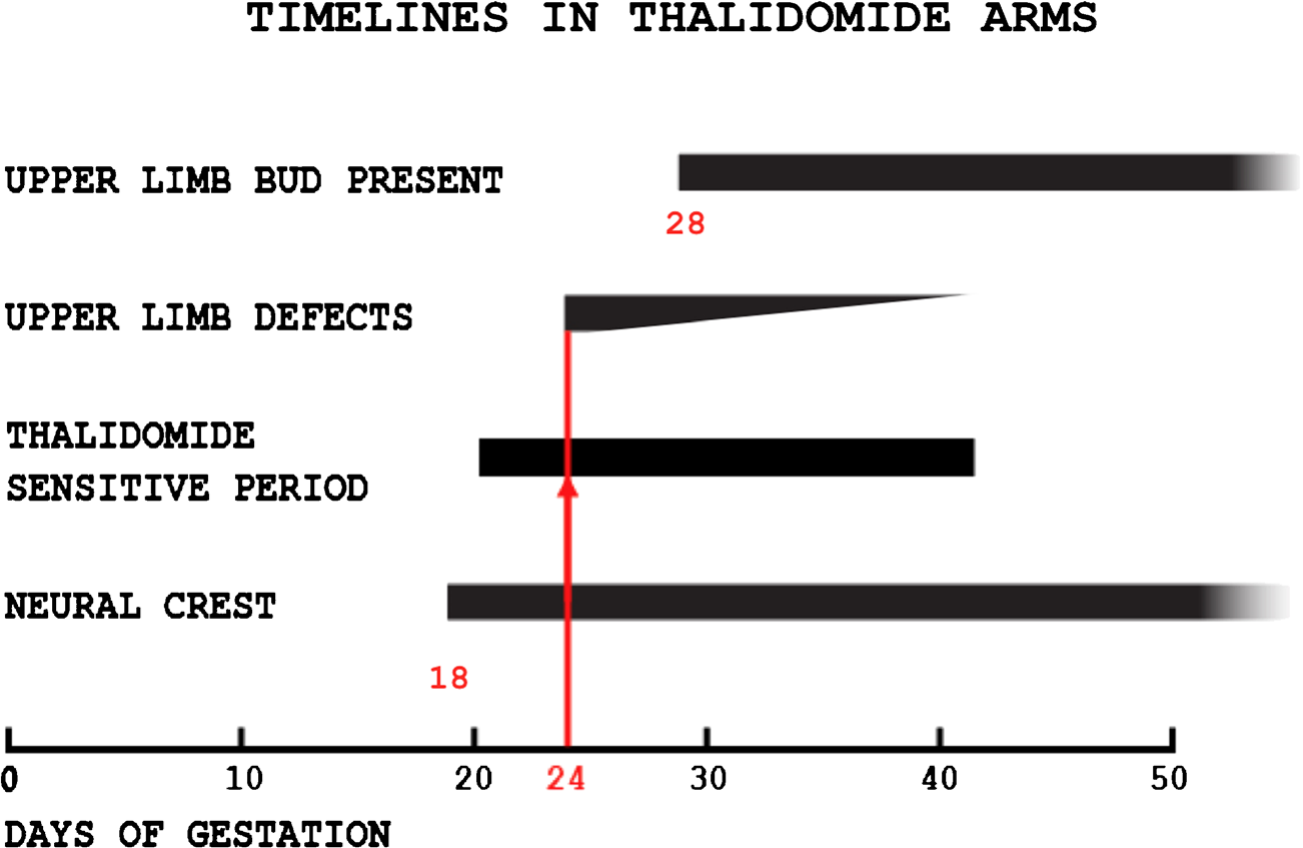
Timelines [4] compiled from Langman [22], Nowack [36], and Knapp et al. [37]. The neural crest appears at day 18 in the human embryo. The upper limb bud appears at day 28 in the human embryo. Thalidomide acts between days 21 and 42. The first arm defects follow ingestion of thalidomide at day 24, when the neural crest is present, but 4 days before the limb bud exists. This was confirmed in laboratory primates by Neubert and Neubert [39]

Additional evidence of neuropathy emerges from timelines of events in early pregnancy [4]. Thalidomide acts between 21 and 42 days of human gestation [36–38], coinciding with evolution of segmental sensory neurons from the neural crest. To cause absence of the radius and thumb, the earliest drug exposure was on the 24th day, 4 days before the upper limb buds first appear on the 28th day (Fig. 7) [36, 37]. Precocious timing of the insult [38] was confirmed in laboratory primates [39], proof that the action of thalidomide predates the limb bud and occurs outside it [39]. That the primary thalidomide injury lies in a nerve, outside the skeleton, explains the histological normality of our skeletal specimens.

Willert and Henkel identified increased mesenchymal mass in patients with polydactyly or triphalangism [2]. The known biphasic reaction of sensory nerves to insult explains this [40]. Clinically, sensory nerve stimulation causes brief initial hypersensitivity (pain, paraesthesia) followed by prolonged sensory suppression (hypoaesthesia, anaesthesia). A brief increase in neurotrophism would increase cell proliferation in that area of nerve supply and generate excess mesenchymal mass, manifesting later as extra phalanges or digits, often adjacent to areas of neurotrophic suppression causing hypoplasia or aplasia of skeletal parts.

The radial/tibial distribution of thalidomide embryopathy may reflect the age of the embryo at the onset of morning sickness. Before thalidomide, German embryologists pondered the question of selective vulnerability [41, 42]: they suggested that recent evolutionary additions to limb function might be more vulnerable to injury than more ancient anatomical structures. Thumb–forefinger opposition (C6) and knee extension to stand upright (L3/4/5) are recent additions. Both segments are prime targets for thalidomide.

A recent study identified peripheral neuropathy in the now middle-aged victims of the thalidomide epidemic [43]. Many survivors have now presented with unexpected late onset sensory neuropathic symptoms (pain, paraesthesia). This “post-thalidomide syndrome” expresses the same principle of axonal loss [4], paralleling “post-polio syndrome”. In both, a major loss of neurons/axons occurs early in life (polio virus/infancy/motor or thalidomide/embryo/sensory). Survivors living with reduced populations of neurons suffer a second loss of axons at middle age—the physiological degeneration of age, normally asymptomatic. But in middle-aged thalidomide survivors with previously damaged peripheral nerves, this second reduction shrinks the neuronal population below the symptom threshold, and symptoms erupt [4, 43]. Their new symptoms are not psychological or simply compressive; they are neuropathic and need to be medically understood and appropriately managed. Thalidomide embryopathy continues to declare itself today as a lifelong, quantitative neurological disorder.

## Conclusion

This unique case collection, gleaned from the thalidomide disaster of 1958–1962, provides a rare insight into the teratogenic mechanism of thalidomide. The absolutely unremarkable microscopic appearance of the mesenchymal components of these digital abnormalities is in stark contrast to the abnormal mass of mesenchyme and the final disordered anatomy.

We confirmed all the findings of Willert and Henkel. They recognised the two opposing processes of thalidomide: either the reduction or addition of mesenchymal mass. Our new discussion explains this biphasic process as neurotoxic damage to embryonic sensory nerves, resulting in the failure of neurotrophism: either suppression of mesenchymal cell proliferation with digital reduction defects, or overstimulation causing excess mesenchyme with polydactyly and extra phalanges. Evidence from clinical medicine and regeneration biology supports the conclusion that thalidomide embryopathy is a quantitative neurological disorder in the early embryo. As in adults, the primary pathology of thalidomide lies outside the skeleton in the sensory nerves supplying embryonic mesenchyme destined for future limb structures. The ultimate skeletal malformations are secondary sensorineural embryonic osteoarthropathies. Neurotrophism is the missing link.
